# An Isolated Case of Unilateral Macro-Ophthalmia With Resultant Anisometropic Amblyopia in Neurofibromatosis 1

**DOI:** 10.7759/cureus.44679

**Published:** 2023-09-04

**Authors:** Fabliha A Mukit, Hays T Cape, Suhaiba S Huq, Shiva Bohn

**Affiliations:** 1 Department of Ophthalmology, Hamilton Eye Institute, The University of Tennessee Health Science Center, Memphis, USA; 2 Ophthalmology, University of Florida, Gainesville, USA

**Keywords:** neurofibromatosis 1, neurofibroma, plexiform neurofibromas, optic pathway glioma, amblyopia, asymmetric macro-ophthalmia, anisometropic amblyopia

## Abstract

The most common causes of vision loss in neurofibromatosis 1 (NF1) patients are sequelae from tumors such as optic pathway glioma, plexiform neurofibroma, or secondary glaucoma. Here we report the case of a six-year-old female with anisometropic amblyopia resulting from an isolated unilateral macro-ophthalmia with a known history of NF1. Our patient progressed to light perception vision in the left eye due to a non-neoplastic cause associated with NF1 with at least two years of documented unilateral macro-ophthalmia without any ophthalmology referral or evaluation. This case aims to highlight the importance of early and deliberate ophthalmologic examination in all patients with neurofibromatosis 1 to assess for appropriate visual development and early intervention.

## Introduction

Neurofibromatosis 1 (NF1) is the most common phakomatosis and neurological disorder caused by a single gene that affects 1 in 3,000 live births worldwide. NF1 is initiated by a de-novo mutation in chromosome 17q11.2 that perpetuates an autosomal dominant inheritance pattern without any sex, racial, geographic, or ethnic predilection [1]. While most NF1 patients have a mild-to-moderate presentation due to the variable expressivity of the mutated neurofibromin, there remains potential for NF1 to cause permanent vision loss, developmental delay, skeletal abnormalities, and benign or malignant tumors in the dermis, brain, or spine [1,2].

Neurofibromin is ubiquitously expressed in the body but has the highest expression in neurons, Schwann cells, oligodendrocytes, and astrocytes [3]. A suboptimal neurofibromin results in the over-functioning of the proto-oncogene Ras, which leads to tumorigenesis. Benign and malignant tumors develop predominantly in the central and peripheral nervous systems [4].

The most reported etiologies for vision loss in NF1 are optic pathway gliomas and plexiform neurofibromas (PN). Plexiform neurofibromas can cause deprivation amblyopia from ptosis, secondary glaucoma from elevated episcleral venous pressure, and refractive amblyopia induced from ptosis or mass effect resulting in astigmatism or anisometropia [3,5-7]. Anisometropic amblyopia has also been reported with asymmetric macro-ophthalmia in cases with plexiform neurofibromas, ocular hypertension or glaucoma, orbito-facial NF1, Francois-Katz syndrome, and optic nerve glioma [5,8].

The present report describes a young patient who presented with acute vision loss and exotropia and failed her school’s vision screening despite yearly evaluations by a pediatrician and neurologist. In this case, we report a rare cause of anisometropic amblyopia from unilateral macro-ophthalmia without any associated tumorous growths from neurofibromatosis 1.

## Case presentation

A 6-year-old, well-appearing African-American female presented to the local pediatric emergency department (ED) with her mother due to concerns of acute vision loss in her left eye. Five days prior to the presentation, her mom noticed the patient struggling with homework and favoring her right eye with intermittent outward drifting of her left eye. When questioned about her behavior, the patient stated she was unable to see anything from her left eye. The patient denied any noticeable vision changes or loss prior to five days ago. Mom denied any other associated symptoms or recent ocular or head trauma. Due to concerns about vision loss, the mom took the patient to her pediatrician, who recommended urgent evaluation by the emergency department.

**Figure 1:**
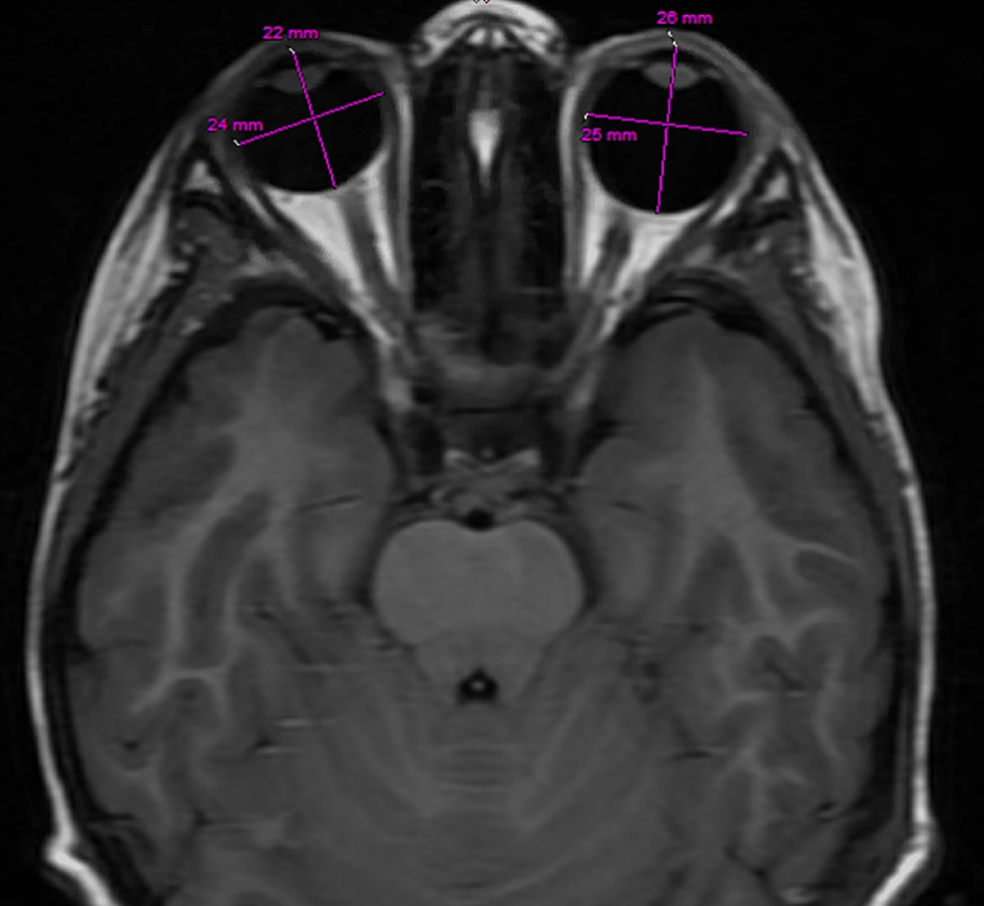
Baseline magnetic resonance imaging of the Brain T1 FLAIR from 2020 showing asymmetric axial elongation to 26 mm of the left eye, compared to 22 mm in the right eye.

The patient's medical history is notable for NF1, which was confirmed at the age of three when she met diagnostic criteria of more than six café-au-lait spots and three subcutaneous neurofibromas [1]. The first MRI brain and orbit with contrast was completed at age four with an isolated finding of left macro-ophthalmia of 26 mm axial length (Figure 1) and no reported optic pathway glioma. The birth, developmental, ocular, family, and social histories were all unremarkable. The patient had never seen any eye care provider prior to consultation with ophthalmology in the ED, and there had been no previous referrals to an eye care provider. The patient did not report any headaches, nausea, vomiting, or poor oral intake. All vital signs and labs acquired in the ED were within normal limits.

**Figure 2:**
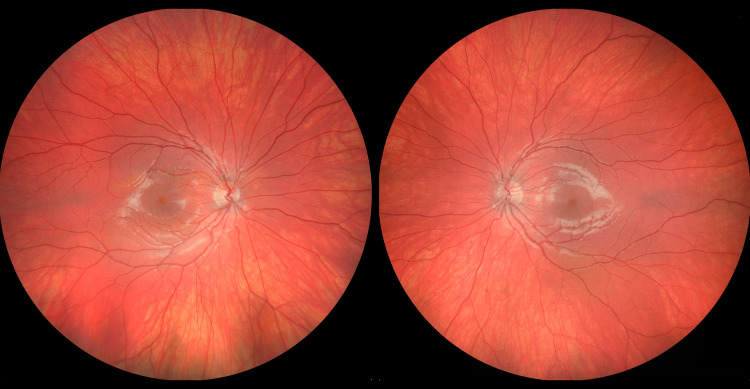
Zeiss Clarus™ fundus photos of the patient with normal posterior pole and optic nerves of both eyes.

**Figure 3:**
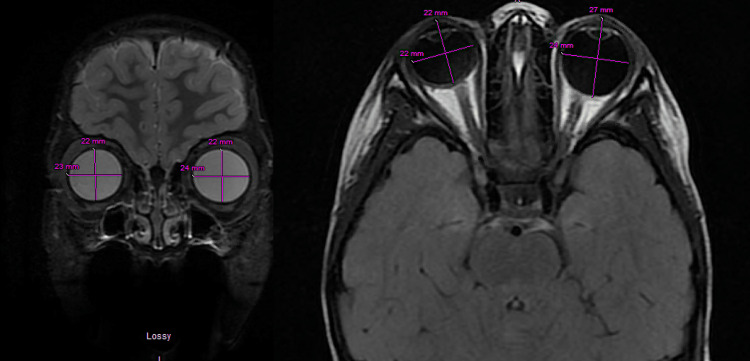
Magnetic resonance imaging of the Brain T1 FLAIR from 2023 showing asymmetric axial elongation to 27 mm of the left eye, compared to 22 mm in the right eye.

On examination by ophthalmology, visual acuity was 20/20 in the right eye (OD) and light perception was 20/20 in the left eye (OS), with a relative afferent pupillary defect in OS. Pupils were equal in size, round and flat, bilaterally. Intraocular pressure was measured as 15 OD and 14 OS with a rebound tonometer. Extraocular movements were normal in both eyes (OU). Margin to reflex distance 1 was 3.5 mm OU, and the globe position was symmetric on Hertel exophthalmometry. Lisch nodules were identified on both eyes, and a dilated fundus exam was unremarkable (Figure 2). MRI brain and orbit with contrast revealed a progressed left macro-ophthalmia, now 27 mm in axial length, with no interval development of an optic pathway glioma (Figure 3).

**Figure 4:**
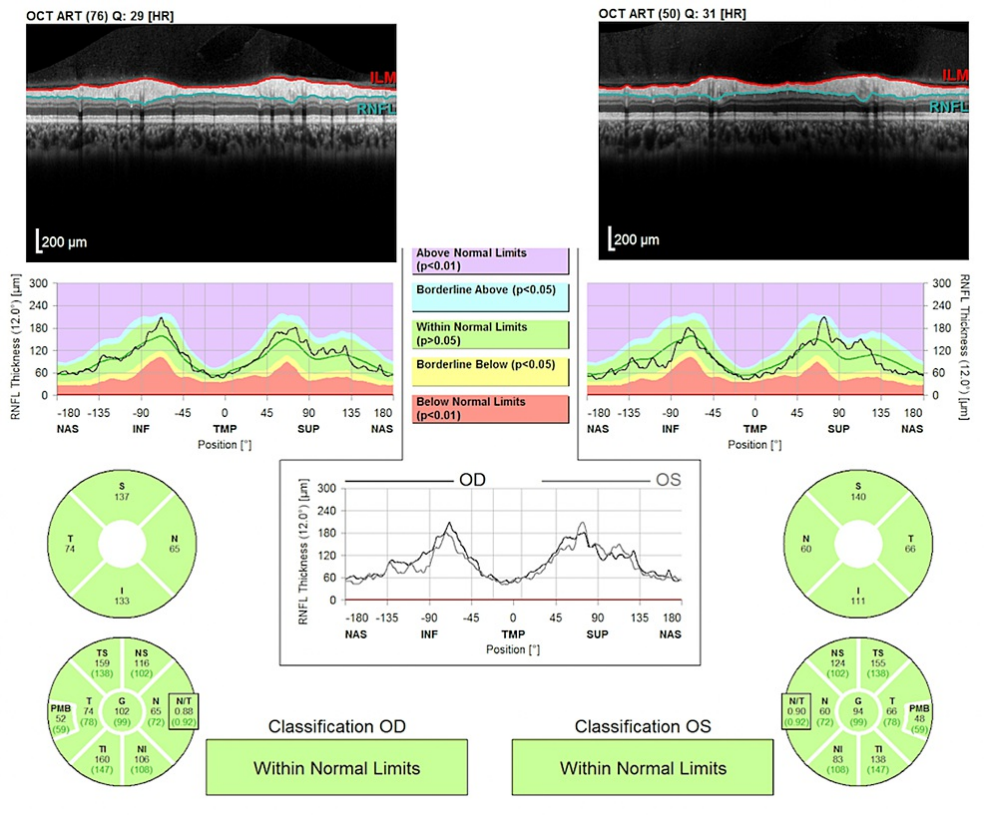
Optical coherence tomography of the retinal nerve fiber layer revealing normal global thickness.

As the child was systemically stable, she was discharged. At her pediatric ophthalmology follow-up two days later, the patient was noted to have stable vision of 20/20 OD and LP OS. Intraocular pressure measured with rebound tonometry was 16 OD and 17 OS. The patient had a positive optokinetic nystagmus at 16 inches upon monocular testing of each eye. Ishihara color plates were 8/10 OD but were unable to be performed on the left eye; the Titmus test was negative; and the Worth 4 dot test results were 4 dots at both near and distance. The corneal diameter was 12.1 OD mm and 12.2 mm OS. Motility measurements revealed orthotropia at near and comitant thirty-prism diopters of exotropia at distance. The cycloplegic refraction of the right eye was Plano, and the left eye was −17.50 +1.50 × 090. Optical coherence tomography of the retinal nerve fiber layer (RNFL) revealed a reliable scan with a global thickness of 102 OD and 94 OS with the full thickness of the RNFL on both eyes, consistent with the dilated fundus examination (Figure 4).

The patient was fitted with trial lenses by the orthoptist and confirmed to tolerate full cycloplegic refraction. Mom chose glasses with balance instead of contact lenses due to hygiene concerns owing to her young age. Extensive counseling was performed regarding the potential for visual improvement in the left eye with good compliance to full-time glasses wear. Additionally, due to her high myopia, Mom was counseled on the risk of retinal detachment, and signs and symptoms with a plan for a dilated fundus examination every six months were discussed. Monocular precautions were reviewed.

At follow-up eight weeks later, the patient’s glasses were confirmed to be Plano OD and −17.50 +1.50 × 090. Vision in the left eye improved enough to count fingers on one foot with a now-resolved intermittent exotropia with compensation. The relative afferent pupillary defect of the left eye was present. As the patient tolerated her new prescription, Mom was recommended to patch the right eye four to six hours a day with full-time glasses and follow up in four months.

## Discussion

In our patient, we report a rare case of an isolated, unilateral macro-ophthalmia in NF1 without any associated plexiform neurofibroma, optic pathway glioma, elevated intraocular pressure, or chorioretinal abnormalities. Our patient has been evaluated by a pediatrician and neurologist annually since birth with no documented visual acuity but reportedly a normal pupillary reaction of both eyes without any APD, full confrontational visual fields, and full extraocular movements. Notably, our patient’s first MRI brain and orbit with contrast at age four revealed a left macro-ophthalmia with an axial length of 22 mm OD and 26 mm OS; however, she did not receive any referral to an eye care provider from her neurologist. In the following years, the right eye's axial length remained stable, and the left eye's increased to 27 mm OS. It is suspected that the vision progressively worsened to at least 20/200 due to severe refractive amblyopia.

Our patient had several consecutive exams where she reported LP vision only; however, the positive optokinetic nystagmus test of the left eye indicates vision better than light perception. However, the left afferent pupillary defect supports the diagnosis of anisometropic amblyopia due to reduced vision in the left eye compared to the right eye. On her consecutive evaluations, the patient did not endorse any neurologic symptoms such as headache, nausea, vomiting, or resultant poor oral intake. Additionally, all imaging of the MRI brain and orbit with and without contrast was reviewed with a radiologist and an ophthalmologist to evaluate for any associated tumors that could have led to this vision loss. There were no visible optic pathway gliomas identified by either service, and the optic nerve caliber and pathway were within normal limits. Additionally, as the patient had normal intra-ocular pressures, corneal diameters, and bilateral global thickness on the RNFL of both eyes (based on age-based normative ranges from several studies), as well as a lack of plexiform neurofibroma of the eyelid, the diagnosis of an isolated, unilateral macro-ophthalmia was made [9-11].

Macro-ophthalmia with resultant refractive error can arise in patients with NF1, and there are several leading theories regarding the pathogenesis. One possible cause is the release of local growth factors from neurofibromas, causing enlargement of the globe [12]. Partial lid occlusion is another potential cause of asymmetric globe growth, as it can disrupt the normal emmetropization process and promote the development of axial myopia. NF1 is also associated with secondary glaucoma. Since young children have a more distensible sclera and cornea, an elevated IOP can cause globe enlargement, an enlarged corneal diameter, and a long axial length [13,14]. In our patient, there was no external finding of a plexiform neurofibroma. The intraocular pressures measured at three consecutive ophthalmology visits were within normal limits. The global RNFL thickness of both eyes had values in the normal range per several pediatric studies [9-11], but there are currently no official pediatric normative values in the OCT software. The normal RNFL was further supported by the dilated fundus exam, which revealed a bilateral cup-to-disc ratio of 0.2 with sharp margins, no deep cupping, and healthy tissue circumferentially.

As NF1 afflictions are heterogeneous, vision loss can often be overlooked by multi-disciplinary management due to inconspicuous changes. Notably, vision changes in children are often undiagnosed until more obvious manifestations, such as recurrent falls or running into walls, occur. An average adult axial length is 23.6 mm, and a single millimeter increase in axial length can cause -2 to 2.5 diopters of myopia [8]. Our patient presented two years ago with at least a 12-diopter myopic shift that later progressed to −17.50 on our refraction. Additionally, our patient also has complete loss of stereopsis, which has been reported to be present in as little as >1 diopter difference in astigmatism and anisometropia [15]. Lack of stereopsis affects the quality of life by limiting the experience of visuospatial depth in scenery and cinema. Even more concerning is the limitation on career options since athletes, surgeons, pilots, and architects all require excellent stereoacuity and visually guided movement, which has been shown to be less accurate and take longer in monocular vision [16]. In our patient, the lack of early referral to an eye care provider has precluded valuable stereopsis rehabilitation, which is also limited by her aniseikonia. Additionally, as the visual development period in a child is finite, the delayed management has impacted the potential for improvement in our patient.

## Conclusions

A subtle change in axial length has significant functional, educational, and social repercussions for a growing child that are easily preventable. Childhood refractive errors can be elusive without deliberate examination, but their impact on lifelong visual health is significant. We share this rare manifestation of NF1 to emphasize the importance of early comprehensive eye examinations for all children. Due to the plasticity of young brains and the lifelong sequelae of amblyopia, careful screening examination by an eye care provider during the period of critical visual development should become the standard of care for all children.
